# Shared Decision-Making and Decision Support in Advanced Chronic Kidney Disease: A Systematic Review of Models, Treatment Pathways, and Patient-Centered Outcomes

**DOI:** 10.7759/cureus.116118

**Published:** 2026-09-11

**Authors:** Awais Naeem, Zulfiqar Ashraf, Sajjad Ali, Dani Badal Hayder Hayder, Vaishali Anil Wahane, Maisha Zaman Poushi, Jedidiah Kolo, Mohannad Jamil Wahdan, Youmna Ibrahim Mohamed Seddik Mohamed Seddik, Rahmeh Riyad Radi Al-maqableh, Sara Alaaldin Mahmoud Alghzawi

**Affiliations:** 1 Emergency Medicine, Central Park Teaching Hospital, Lahore, PAK; 2 Urology, Virgen Milagrosa University Foundation, San Carlos, PHL; 3 Urology, Ipswich Hospital NHS Trust, Ipswich, GBR; 4 Internal Medicine, Khairpur Medical College, Khairpur, PAK; 5 Internal Medicine, College of Medicine, University of Duhok, Duhok, IRQ; 6 Radiology, Ultrascan Diagnostic Centre, Mumbai, IND; 7 Emergency Medicine, Dhaka Medical College and Hospital, Dhaka, BGD; 8 Emergency Medicine, Tameside and Glossop Integrated Care NHS Foundation Trust, Ashton-under-Lyne, GBR; 9 Endocrinology, University of Debrecen Medical School, Debrecen, HUN; 10 Endocrinology, Addenbrooke's Hospital, Cambridge, GBR; 11 Medicine, University of Debrecen Medical School, Debrecen, HUN; 12 Internal Medicine, University of Debrecen Medical School, Debrecen, HUN; 13 Internal Medicine, Yarmouk University, Irbid, JOR

**Keywords:** chronic kidney disease (ckd), conservative kidney management, dialysis modality choice, patient-centered care, shared decision-making

## Abstract

Shared decision-making (SDM) is a crucial part of patient-centered care because advanced chronic kidney disease (CKD) requires complex treatment choices. This study systematically reviews models of SDM and decision-support interventions in advanced CKD, focusing on treatment pathways, modality selection, and patient-centered outcomes. We conducted a systematic review in accordance with accepted methodological guidelines. From the beginning to May 2026, we searched PubMed, Embase, the Cochrane Library, and Scopus. We included studies assessing SDM models, treatment routes, decision-support treatments, and patient-centered outcomes in advanced CKD. We used the Joanna Briggs Institute (JBI) critical appraisal tools for cross-sectional studies, the Risk of Bias in Nonrandomized Studies of Interventions (ROBINS-I) tool for nonrandomized studies, and the Risk-of-Bias Assessment Tool 2.0 (RoB 2.0) for randomized trials. We included seven studies involving 3,130 participants. The mean or median participant age ranged from 60.8 to 82 years, and female representation ranged from 38% to 50%. SDM quality was moderate (9-item Shared Decision-Making Questionnaire (SDM-Q-9): 52.5 ± 27.8/100) and improved with alternative education (β = 8.2; 95% CI: 0.7-15.7; p = 0.03). Treatment preference concordance was significant, with 68% choosing home-based dialysis and 83% starting their preferred modality. SDM was associated with lower odds of unscheduled dialysis initiation (33.3% vs. 66.7%; adjusted OR = 0.19; 95% CI: 0.07-0.47; p = 0.001) and increased peritoneal dialysis (PD) uptake (27.3% overall; 27.27% vs. 13.33% in conventional care). Decision-aid use increased patient-family agreement from κ = 0.25 to κ = 0.63. While knowledge of conservative kidney treatment was only 7% at baseline, supportive care knowledge rose from 2.02 ± 0.96 to 2.36 ± 0.93 (p < 0.01). Additionally, 76.2% of participants preferred early disclosure of kidney failure risk (p = 0.03). Frailty (HR = 6.42; p = 0.001) and BMI <25 kg/m² (HR = 2.84; p = 0.01) were associated with increased mortality. Intervention acceptability was high, with 98% satisfaction, 96% perceived usefulness, and 52.2% spending >20 minutes using decision-support tools. In advanced CKD, SDM and decision-support interventions may improve treatment alignment, patient understanding, involvement, and care planning, supporting their potential incorporation into standard nephrology practice.

## Introduction and background

Advanced chronic kidney disease (CKD) represents a challenging stage in a patient’s care journey, during which patients and clinicians face multiple treatment options: hemodialysis, peritoneal dialysis (PD), kidney transplantation, and conservative kidney management (CKM) [1]. Each modality has its own advantages and disadvantages for survival, lifestyle disruption, symptom burden, and health care utilization, and a thoughtful discussion of individual values and preferences is required [2]. Shared decision-making (SDM) is considered the “gold standard” for decisions in which preferences matter, as it involves two-way information sharing, risk communication, and co-creating goals [3].

In nephrology, however, SDM is not consistently implemented because time is often limited, patient-friendly decision support tools are lacking, and prognosis is uncertain [4]. Decision aids, which can take the form of an educational booklet, video, or interactive web-based platform, have been shown to enhance knowledge, decrease decisional conflict, and help patients make decisions aligned with their values in a variety of medical situations [5].

However, information on advanced CKD remains dispersed, with differences in SDM models, treatment approaches, and outcome assessment [6]. Furthermore, compared with clinical outcomes, patient-centered outcomes such as treatment concordance, psychosocial well-being, and quality of life are frequently underreported [7]. In this review, SDM process and quality outcomes assess the extent and quality of patient involvement in treatment decisions. In contrast, treatment preference and modality uptake reflect whether patients’ informed preferences translate into actual treatment choices. Patient knowledge, awareness, and beliefs assess whether decision-support interventions improve understanding of available options and prognosis. Clinical safety and survival outcomes capture the potential clinical consequences of treatment decisions, whereas tool feasibility and acceptability assess whether decision-support interventions are practical, engaging, and acceptable to patients. Together, these outcomes provide a broader assessment of decision quality by considering whether decisions are informed, preference-concordant, clinically appropriate, and acceptable to patients. To identify effective techniques and gaps that need more research, this systematic review synthesizes the available data on SDM models and decision support interventions in advanced CKD, analyzing their effects on treatment pathways, patient knowledge, decision quality, and clinical safety.

## Review

This systematic review followed the Preferred Reporting Items for Systematic Reviews and Meta-Analyses (PRISMA) 2020 principles to provide transparent, comprehensive, and methodologically robust reporting [8]. The review protocol was prospectively entered into the International Prospective Register of Systematic Reviews (PROSPERO) database (registration number). The Cochrane Handbook for Systematic Reviews of Interventions (version 6.5) guided the approach to ensure that best-practice evidence synthesis standards were followed (CRD420261421853) [9].

Study selection process and search strategy

A comprehensive and systematic literature search was carried out in the following electronic databases: PubMed/MEDLINE, Scopus, and the Cochrane Central Register of Controlled Trials. The search included studies from database inception to May 2026 and was limited to English-language publications.

The search approach combines Medical Subject Headings (MeSH) with free-text terms such as shared decision-making, decision support tools, and advanced CKD. Key terms included versions such as “shared decision making,” “decision aid,” “decision support system,” “advanced chronic kidney disease,” “end-stage renal disease,” “dialysis decision-making,” and “kidney transplantation decision pathways.”

Boolean operators (AND, OR) were utilized to improve search sensitivity and specificity. Furthermore, we manually screened reference lists from included studies, pertinent systematic reviews, and meta-analyses to identify additional suitable papers not found through database searches. The Supplementary Appendix (Table 5 in Appendices) provides a detailed search strategy for each database.

Eligibility Criteria

Studies were selected according to predefined eligibility criteria based on population, intervention, outcomes, study design, and publication characteristics. Eligible studies included adults with advanced CKD or end-stage kidney disease and evaluated SDM interventions in treatment planning. Studies were required to report at least one relevant patient-centered outcome related to SDM and employ an eligible study design. We excluded studies involving pediatric populations, non-CKD conditions, non-SDM interventions, or those lacking extractable outcome data. Table 1 provides a detailed summary of the inclusion and exclusion criteria.

**Table 1:** Predefined eligibility criteria for included studies CKD: chronic kidney disease; ESKD: end-stage kidney disease; SDM: shared decision-making

Domain	Inclusion criteria	Exclusion criteria
Population	Adults aged ≥18 years with advanced CKD (stage 4-5) or ESKD facing treatment decisions	Pediatric populations (<18 years); studies focused on acute kidney injury or non-CKD conditions
Intervention/concept	Studies evaluating SDM approaches, including decision aids, communication frameworks, counseling interventions, patient–provider decision tools, or formal SDM models	Studies not evaluating SDM or decision-support interventions
Outcomes	Studies reporting at least one SDM-related outcome, including participation in decision-making, decisional conflict/regret, understanding of treatment options, treatment choice, patient experience/satisfaction, or quality of communication	Studies without extractable outcomes relevant to the review objectives
Study design	Randomized controlled trials, quasi-experimental studies, cohort studies, cross-sectional studies, and qualitative studies	Case reports, editorials, narrative reviews, conference abstracts with insufficient data, and preclinical studies.
Publication characteristics	Peer-reviewed full-text articles published in English	Duplicate publications (unless the most comprehensive report was available), non-English publications, and articles without full-text availability

Data Extraction

To maintain uniformity and reduce errors, two reviewers extracted data separately using a consistent, predesigned extraction form. Extracted variables comprised study parameters (design, setting, sample size, and participant demographics), as well as SDM-related outcomes. Data were divided into five categories: SDM method and quality, knowledge and awareness, treatment choices, clinical results, and feasibility/acceptability measures. We resolved disagreements through discussion or by consulting a third reviewer.

Quality Assessment

We evaluated the methodological quality of the included studies using validated, study-design-specific techniques to ensure a thorough assessment of internal validity and risk of bias across evidence categories. We used the Cochrane Risk of Bias Assessment Tool 2.0 (RoB 2) method to assess bias in five domains: the randomization procedure, deviations from intended interventions, missing outcome data, outcome assessment, and selective reporting. Each domain was rated as low risk, some concerns, or high risk of bias, and an overall risk-of-bias assessment was made based on the highest level of concern identified in every domain [9].

Methodological quality in cross-sectional and observational noninterventional studies was assessed using the Joanna Briggs Institute (JBI) critical assessment checklists tailored to the study design. These tools evaluated domains such as sampling technique, measurement validity, confounding variables, and statistical analysis [10].

The ROBINS-I tool was used for nonrandomized intervention trials and retrospective cohort studies. This instrument assessed bias from confounding, participant selection, intervention classification, deviations from intended interventions, missing outcome data, outcome measurement, and selective reporting. Each study was assigned a low, moderate, severe, or critical risk of bias [11]. Two reviewers conducted all assessments independently, and they resolved disputes through discussion or by consulting a third reviewer.

Results

Study Selection Process

We searched four databases (PubMed, Embase, Scopus, and Cochrane) and found 755 records. We reviewed 521 records and eliminated 150 after removing 234 duplicates. Nine of the 371 reports whose full texts were evaluated could not be recovered. Seven studies were ultimately included in the final review after eliminating research with incorrect interventions, populations, outcomes, and conference abstracts (Figure 1).

**Figure 1:**
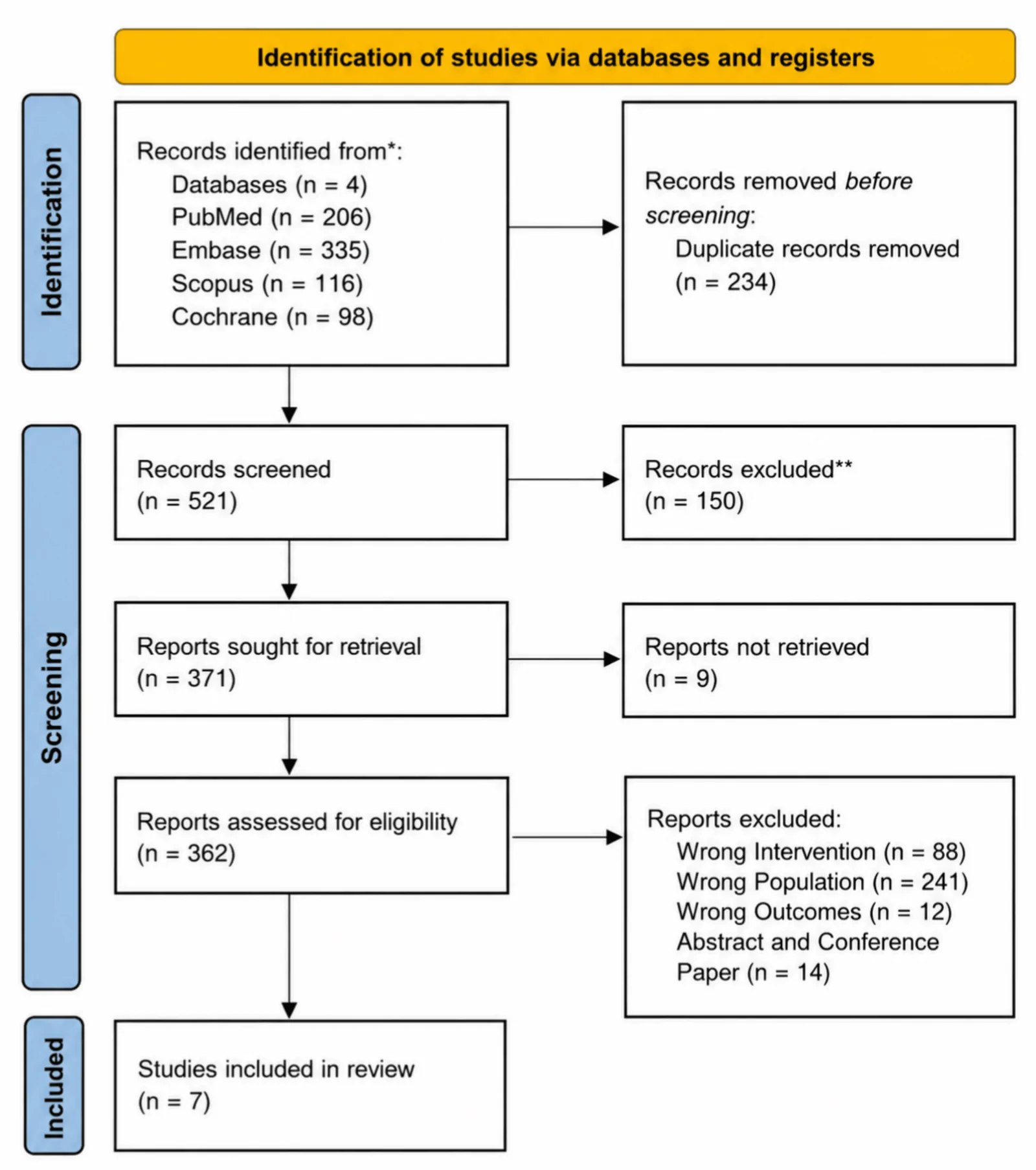
PRISMA flow diagram PRISMA: Preferred Reporting Items for Systematic Reviews and Meta-Analyses *Records identified from the electronic databases searched, including PubMed, Embase, Scopus, and the Cochrane Library. ** Records excluded during title and abstract screening because they did not meet the predefined eligibility criteria Source: Adapted from [8]

Study Characteristics and Baseline Demographics

Seven studies [12-18], with different study designs including randomized controlled trials, cross-sectional analyses, cohort studies, and qualitative studies, were included. These studies ranged from small single-center studies (42-1,957 subjects). The reported mean or median participant age ranged from 60.8 to 82 years, with one study reporting that 51% of participants were older than 60 years, highlighting a mostly elderly CKD population. The proportion of females ranged from 38% to 50% among the studies. In Western countries, most cohorts were predominantly White (66% to 90%); in US studies, Black participants constituted up to 31% of the study population. In contrast, Asian participants were reported in UK cohorts. The clinical population was mostly advanced CKD (CKD, stage 4-5 or equivalent), with severely diminished renal function (median eGFR: 13-30 mL/min/1.73m²). There was consistent reporting of high comorbidity burden, with diabetes reported in up to 48% of cases, hypertension in up to 100% in one cohort, and high Charlson Comorbidity Index scores (median up to 7). Several studies explicitly recruited patients using a range of treatment processes to make decisions about dialysis initiation, dialysis modality, or CKM (Table 2).

**Table 2:** Study characteristics and baseline demographics CKD: chronic kidney disease; eGFR: estimated glomerular filtration rate; CCI: Charlson Comorbidity Index; IQR: interquartile range; RCT: randomized controlled trial; USA: United States of America; UK: United Kingdom; y: years Baseline clinical status is reported as presented in the original studies. Values are expressed as mean ± standard deviation, median (IQR), median (range), or percentage, as appropriate

Author (year)	Study design	Setting	Sample size (N)	Age (mean/median)	Gender (% female)	Race/ethnicity (main)	Baseline clinical status
Eneanya et al. (2020) [12]	Parallel-arm RCT	Nephrology clinics (Boston & Philadelphia, USA)	100	Median 75y (IQR 70-81)	49%	66% White, 31% Black	eGFR < 30 mL/min; median CCI = 7
Frazier et al. (2022) [13]	Cross-sectional observational	4 US cities (Boston, Chicago, San Diego, Portland)	350	77.6 ± 5.8y	42%	78% White, 13% Black	87% stage 4-5 CKD; 48% diabetes
O’Keeffe et al. (2025) [14]	Longitudinal retrospective cohort	Salford Royal Hospital, UK	1,957	Median 69y (IQR 57-78)	42%	82% White, 13% Asian	Median eGFR 16; 100% hypertension; 45% diabetes
Mongkolrattanakul (2025) [15]	Retrospective cohort	Phanatnikhom Hospital, Thailand	111	51% > 60y	45%	Thai (location-based)	Stage 4-5 CKD
de-la-Motte (2025) [16]	Longitudinal observational cohort	Denmark (regional + university hospitals)	484	Median 70y (range 18-90)	38%	90% ethnic Danish	Median eGFR 13; median CCI = 5
Prakash et al. (2026) [17]	Qualitative cross-sectional	Single US center	42 patients/11 providers	60.8 ± 14.5y (patients)	50%	69% White, 21% Black	CKD stages 3b–5 (decided/undecided/urgent)
Wong et al. (2023) [18]	Randomized pilot trial	4 medical centers (Seattle, USA)	86 patients/53 family members	82 ± 6y (patients)	44%	83% White	CKD stage 4-5

RoB 2.0

The RoB 2.0 assessment rated Eneanya et al. as at overall high risk of bias, mainly due to concerns about outcome measurement and deviations from intended interventions, despite low risk in the randomization and selection domains [12]. In contrast, Wong et al. reported an overall risk of bias of “some concerns,” with generally low risk in most domains but uncertainty in intervention adherence and outcome measurement. Overall, the evidence base shows predominantly moderate methodological quality, with variability across domains, particularly in outcome assessment bias (Figures 2-3) [9].

**Figure 2:**
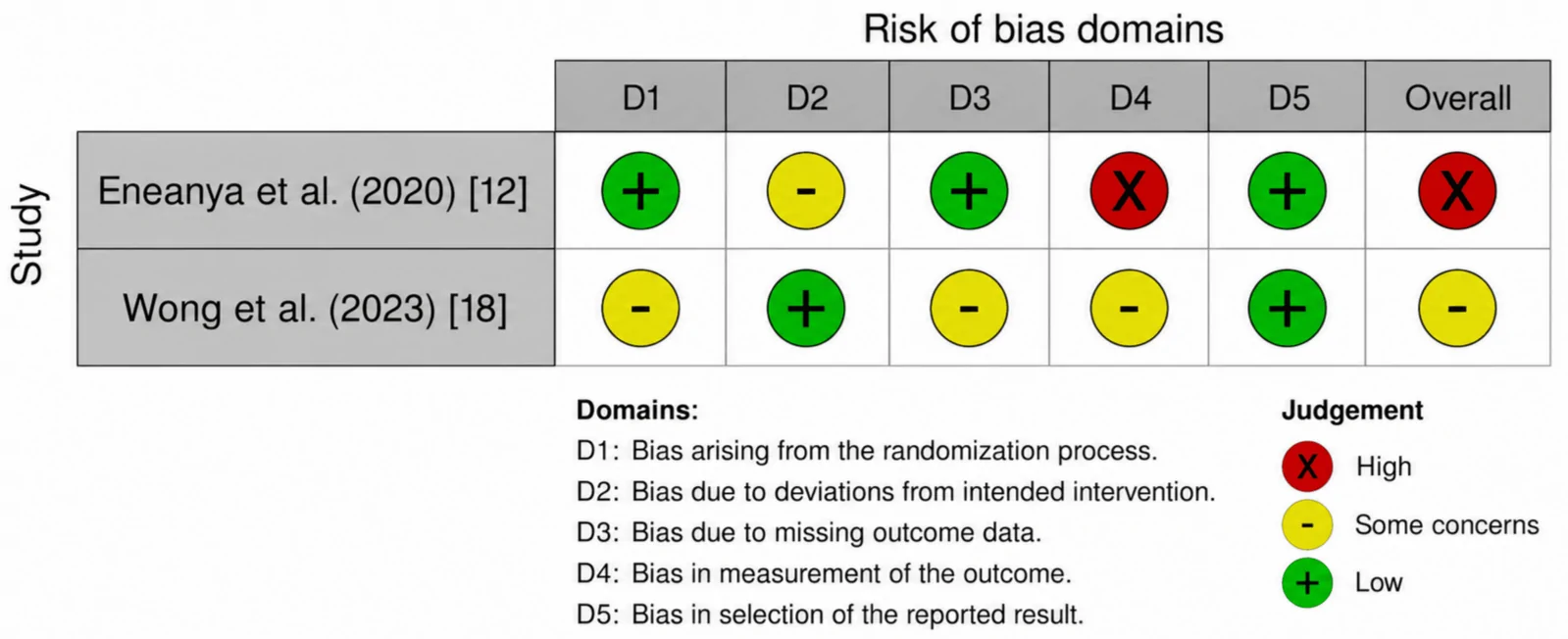
Traffic light plot showing risk of bias for included studies using ROB 2.0 ROB 2.0: Risk-of-Bias Assessment Tool 2.0 Traffic light plot showing domain-level and overall risk-of-bias judgments for each included study. D1 = bias arising from the randomization process; D2 = bias due to deviations from intended interventions; D3 = bias due to missing outcome data; D4 = bias in measurement of the outcome; D5 = bias in selection of the reported result. Green indicates low risk of bias, yellow indicates some concerns, and red indicates high risk of bias Source: Adapted from [9]

**Figure 3:**
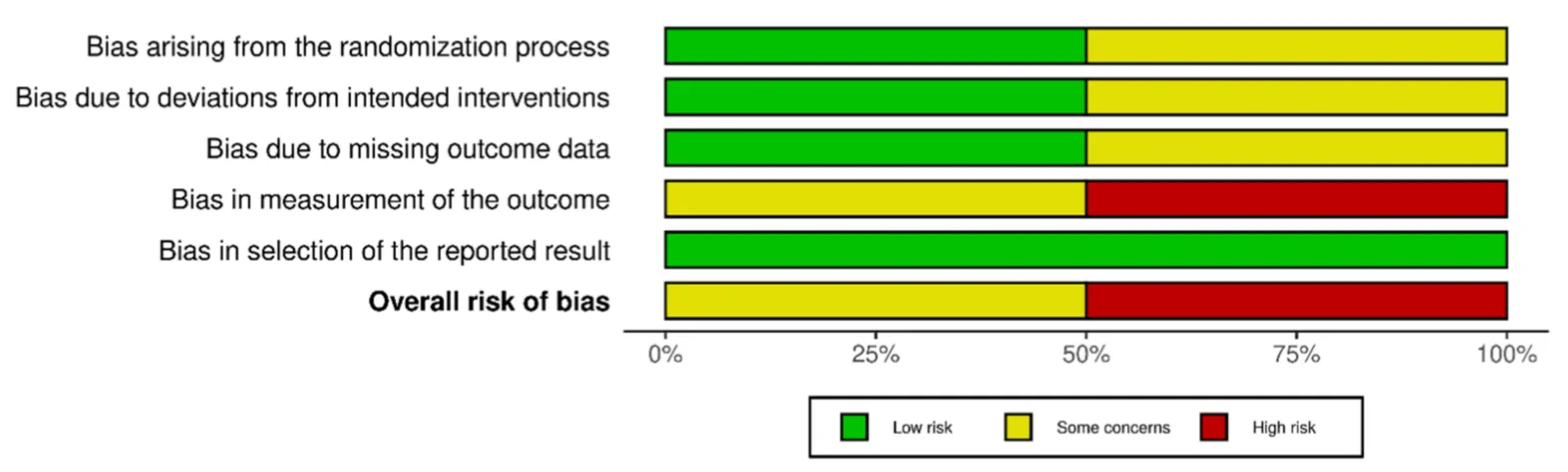
Traffic light plot summary for included studies using ROB 2.0 ROB 2.0: Risk-of-Bias Assessment Tool 2.0 Green indicates low risk of bias, yellow indicates some concerns, and red indicates high risk of bias. The horizontal stacked bars represent the percentage of included studies classified within each risk-of-bias category across the ROB 2.0 domains and the overall risk-of-bias assessment Source: Adapted from [9]

ROBINS-I

The ROBINS-I assessment showed that Mongkolrattanakul et al. had an overall moderate risk of bias, mainly due to moderate concerns about confounding [15]. de-la-Motte et al. were similarly deemed to have an overall moderate risk of bias, with serious risk of bias due to confounding and moderate risk due to missing data. O’Keeffe et al., in contrast, were rated as having an overall serious risk of bias, primarily due to serious concerns regarding confounding. All other ROBINS-I domains across all studies (selection of participants, classification of interventions, deviations from intended interventions, measurement of outcomes, and selection of reported results) were found to be at low risk of bias (Figures 4-5) [10].

**Figure 4:**
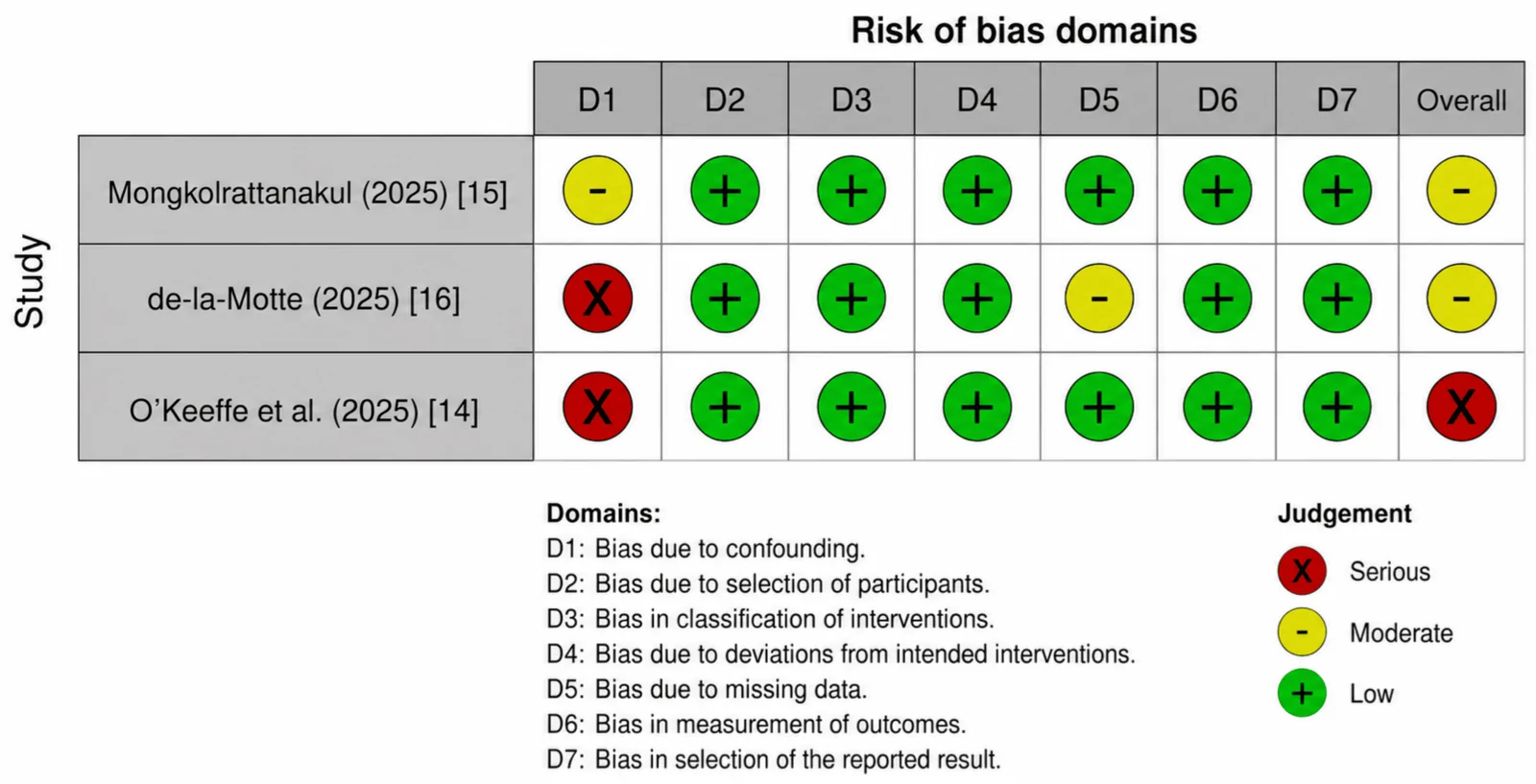
Traffic light plot showing risk of bias for included studies using ROBINS-I ROBINS-1: Risk of Bias in Nonrandomized Studies of Interventions Green (+): low risk of bias; yellow (−): moderate risk of bias; red (×): serious risk of bias. D1: bias due to confounding; d2: bias due to selection of participants; D3: bias in classification of interventions; D4: bias due to deviations from intended interventions; D5: bias due to missing data; D6: bias in measurement of outcomes; D7: bias in selection of the reported result. Overall judgments are shown in the final column Source: Adapted from [11]

**Figure 5:**
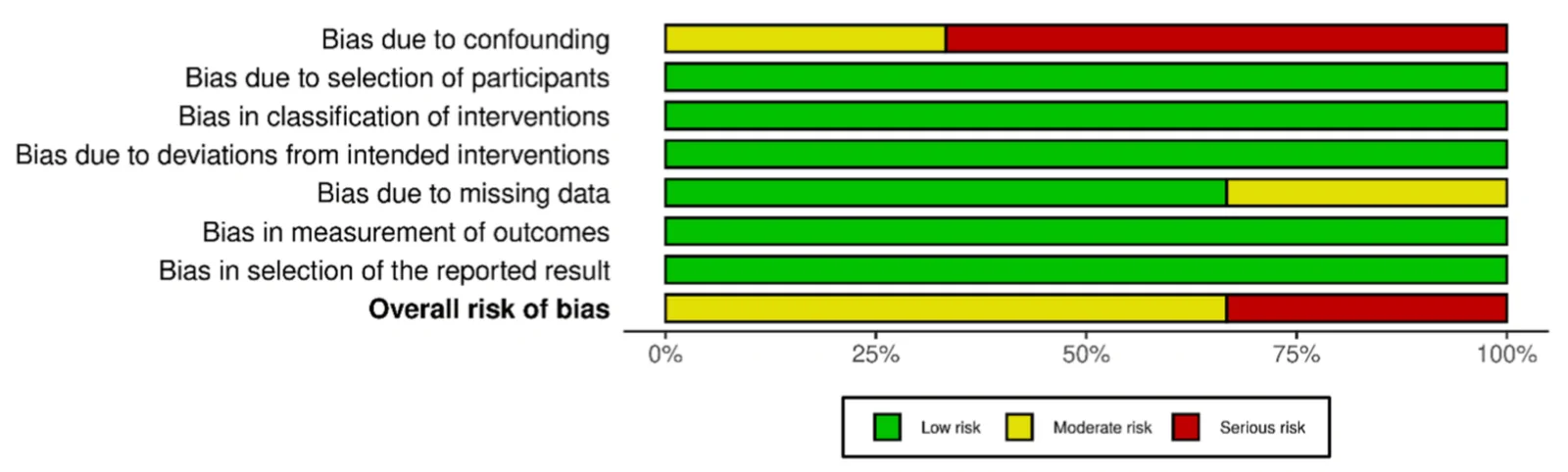
Traffic light plot summary for included studies using ROBINS-I ROBINS-1: Risk of Bias in Nonrandomized Studies of Interventions Green indicates low risk of bias, yellow indicates moderate risk of bias, and red indicates serious risk of bias. The horizontal stacked bars represent the percentage of included studies classified within each risk-of-bias category across individual ROBINS-I domains and the overall risk-of-bias assessment Source: Adapted from [11]

Joanna Briggs Institute (JBI) Critical Assessment Checklists for Cross-Sectional Studies

The JBI critical appraisal indicated that Frazier et al. [13] achieved high methodological quality, with a perfect score of 9 out of 9, showing low risk of bias in all domains. Prakash et al. [17], on the other hand, showed moderate quality (7 out of 9), with one “No” and one “Unclear,” signaling concerns about methodological clarity and reporting. Overall, the cross-sectional evidence showed good quality, with only minor methodological limitations in one study (Table 3) [11].

**Table 3:** Risk-of-bias assessment of included studies using the Joanna Briggs Institute (JBI) Critical Appraisal Checklist for cross-sectional studies JBI: Joanna Briggs Institute Checklist items: (1) clearly defined inclusion criteria; (2) detailed description of study subjects and setting; (3) valid and reliable measurement of exposure; (4) objective and standard criteria used for measurement of the condition; (5) identification of confounding factors; (6) strategies to deal with confounding factors; (7) valid and reliable measurement of outcomes; (8) appropriate statistical analysis; (9) adequate response rate and management of low response rate Source: Adapted from [10]

Study ID	Checklist									Total score
	1	2	3	4	5	6	7	8	9	
Frazier et al. (2022) [12]	Yes	Yes	Yes	Yes	Yes	Yes	Yes	Yes	Yes	9
Prakash et al. (2026) [17]	Yes	Yes	No	Yes	Yes	Yes	Yes	Yes	Unclear	7

Outcomes

The primary and secondary outcomes are summarized in Table 4.

**Table 4:** Summary of primary and secondary outcomes in shared decision-making in advanced CKD SDM: shared decision-making; CKD: chronic kidney disease; SDM-Q-9: 9-item Shared Decision-Making Questionnaire; PD: peritoneal dialysis; CKM: conservative kidney management; KFRE: kidney failure risk equation; HR: hazard ratio; OR: odds ratio; aOR: adjusted odds ratio; β: regression coefficient; κ: Cohen's kappa coefficient; NR: not reported

Author (Year)	SDM quality/process	Knowledge & awareness	Treatment preference/modality	Clinical outcomes	Feasibility & acceptability
Eneanya et al. (2020) [12]	NR	Knowledge ↑ 2.02 ± 0.96 to 2.36 ± 0.93 (p < 0.01)	Preference for supportive care: 26% (p = 0.49)	NR	98% satisfied; 96% found video helpful
Frazier et al. (2022) [13]	SDM-Q-9 score: 52.5 ± 27.8	NR	NR	NR	Options class: β = 8.2 (p = 0.03); Black race: β = 9.6 (p = 0.04)
O’Keeffe et al. (2025) [14]	NR	NR	PD 27.3%; Pre-emptive transplant 15.5%	5-year survival 49.6%; HR (≥80 vs <50) = 10.5	NR
Mongkolrattanakul (2025) [15]	SDM reduces unplanned dialysis	NR	PD protective (OR = 0.26)	Unplanned dialysis: 33.3% vs 66.7% (p < 0.001); aOR = 0.19	NR
de-la-Motte (2025) [16]	Treatment concordance 83%	NR	Home dialysis choice 68%	12% died before initiation	NR
Prakash et al. (2026) [17]	NR	KFRE disclosure preference 76.2% (p = 0.03)	NR	NR	76.2% requested grief support; 42.9% peer support
Wong et al. (2023) [18]	κ = 0.63 vs 0.25 (agreement)	Low CKM awareness (7%)	CKM discussion: 26.4% vs 3.0% (p = 0.007)	NR	21.4% attrition; high engagement

Primary Outcomes

SDM process and quality: Four studies [13,16-18] assessed the quality and efficacy of SDM in advanced CKD. Frazier et al [13] found that patients assessed SDM quality as moderate, with a mean SDM-Q-9 score of 52.5 ± 27.8 (range: 0-100). Higher SDM scores were strongly associated with attendance at an alternative education class (β = 8.2; 95% CI: 0.7-15.7; p = 0.03) and Black race (β = 9.6; 95% CI: 0.6-18.6; p = 0.04). According to de-la-Motte et al. [16], 83% of patients commenced their preferred treatment method, indicating a high degree of congruence between patient preference and actual treatment. Wong et al. discovered that using a decision aid enhanced patient-family agreement, from κ = 0.25 (fair agreement) in standard care to κ = 0.63 (strong agreement) in the intervention group. Attrition rates in both trial arms were 21.4% (p = 1.00). According to Prakash et al. [17], 76.2% (32/42) of participants required extra shock and grief help, while 42.9% (18/42) asked for peer support, indicating severe psychological requirements within SDM pathways.

Treatment preference and modality uptake: Three studies [14-16] examined treatment preferences and modality selection. According to O’Keeffe et al., the most common initial modality choices were PD at 27.3%, pre-emptive kidney transplantation at 15.5%, and alternative options such as conservative approaches [14]. Mongkolrattanakul et al. reported higher PD use in SDM-based care than in conventional care (27.27% vs. 13.33%) [15]. SDM significantly decreased unscheduled dialysis initiation (33.3% vs. 66.7%; p < 0.001), with an adjusted OR of 0.19 (95% CI: 0.07-0.47; p = 0.001). Furthermore, PD was independently protective against unscheduled dialysis (OR = 0.26; 95% CI: 0.07-0.85; p = 0.032). According to de-la-Motte et al., 68% of patients chose home-based dialysis after SDM, and 83% commenced their chosen modality, demonstrating strong alignment between preferences and treatment delivery [16].

Secondary Outcomes

Patient knowledge, awareness, and beliefs: Three studies assessed patients’ knowledge and awareness [12,17,18]. Eneanya et al. found a significant improvement in supportive care knowledge, rising from 2.02 ± 0.96 to 2.36 ± 0.93 (p < 0.01) [12]. After the intervention, 26% of participants chose supportive care (p = 0.49 versus baseline), 98% were happy, and 96% found the film useful. Wong et al. found limited baseline awareness of conservative kidney care, with only 7% of patients aware of this option, indicating significant informational gaps [18]. Prakash et al. found that 76.2% of participants supported early disclosure of kidney failure risk equation (KFRE) results, which was strongly related to education level (p = 0.03) [17].

Clinical safety and survival: Three studies reported clinical outcomes [14-16]. Mongkolrattanakul et al. showed significantly lower unplanned dialysis initiation in SDM care (33.3% vs. 66.7%; p < 0.001), with an adjusted OR of 0.19 (95% CI: 0.07-0.47; p = 0.001) [15]. O’Keeffe et al. reported a five-year overall survival of 49.6%, with survival highest in pre-emptive transplant patients (92.3%), followed by PD [14]. In a study by de-la-Motte et al., patients aged ≥80 years had a significantly higher risk of mortality compared to those under 50 years (HR = 10.5, p < 0.001) [16]. Frailty (HR = 6.42, p = 0.001) and BMI <25 kg/m² (HR = 2.84, p = 0.01) were identified as significant predictors of mortality.

Tool feasibility and acceptability: Two studies examined feasibility and acceptability [12,18]. Wong et al. discovered that patients who were given a decision aid were substantially more likely to discuss treatment alternatives with doctors (26.4% vs. 3.0%; p = 0.007) and family members (26.9% vs. 0%)[18]. A total of 52.2% of users spent more than 20 minutes evaluating the instrument, indicating a high level of engagement. Eneanya et al. observed very high acceptability, with 98% of participants satisfied and 96% rating the intervention as useful [12].

Discussion

This systematic analysis shows how SDM and decision-support interventions may enhance a range of patient-centered outcomes in advanced CKD. Overall, the included studies demonstrated more participation in treatment discussions, greater alignment between patients' chosen and received treatments, increased consideration of home-based dialysis, enhanced knowledge, and increased communication with healthcare providers and family members. The studies also revealed significant gaps in awareness of CKM, emphasizing the need for psychological and peer support. Overall, these findings suggest that SDM can help patients make better-informed, preference-concordant decisions rather than simply directing them to a specific treatment modality. This interpretation aligns with the current nephrology literature, which emphasizes SDM as an active, iterative process in renal failure management [19].

Furthermore, our study discovered that SDM increased patients' engagement in treatment decisions while also strengthening agreement between patients and their families on treatment options. These findings are consistent with an earlier study, which has shown that patients with severe CKD frequently want more engagement in decision-making yet may remain passive participants in dialysis conversations [20]. Previous qualitative research has also shown that individual values, autonomy, lifestyle, emotions, family support, and the quality and timeliness of education all affect dialysis decisions [21]. Thus, the current findings emphasize the need to shift from simply presenting information to a collaborative approach in which doctors actively explore patients' goals and preferences. However, the moderate quality of SDM reported in one included study suggests that implementation is still incomplete. Communication and patient participation are also identified as areas for development in the current nephrology literature [20]. SDM should therefore begin before dialysis becomes necessary and be reviewed when patients' clinical conditions and preferences change.

Notably, the review indicated increased consideration and adoption of home-based dialysis, particularly PD, within SDM pathways, as well as high agreement between patients' desired and delivered modalities. This is consistent with earlier evidence that structured patient education can lead to more informed decisions about home dialysis. Studies have found that patient education is associated with increased selection and receipt of PD, but other research has found that comprehensive predialysis education can improve informed home-dialysis decisions [22,23]. Importantly, these findings do not imply that SDM automatically promotes PD. Previous studies show that patients select dialysis modalities based on independence, flexibility, lifestyle, treatment load, and available support [21,24]. As a result, higher PD adoption may reflect better identification of patient preferences rather than treatment-directed counseling. The primary goal of SDM remains to ensure that patients understand all clinically appropriate alternatives and choose the option that best aligns with their beliefs.

Another significant finding of this review was increased patient awareness. However, the low initial knowledge of CKM shows that many patients would not be given a truly complete option between conservative management and kidney replacement therapy. Earlier studies also found inadequate education and understanding to be significant obstacles to making well-informed dialysis decisions [21,25]. According to a human-centered design study of a CKM decision aid, most patients were initially unfamiliar with treatment options other than dialysis, highlighting the need to present CKM openly during education [26]. Moreover, these findings have significant ramifications because, even when many therapies are theoretically available, SDM can be limited by a lack of understanding. Decision aids can help patients understand complex options and consider how each strategy aligns with their personal goals. Decision aids can enhance understanding, facilitate treatment discussions, and lessen decisional uncertainty, according to data from randomized and implementation studies [27]. Rather than being limited to the time just before dialysis begins, education should start early and be repeated over time.

Additionally, the included evidence suggests a decrease in unplanned dialysis initiation, indicating a potential clinical benefit of structured SDM. Similarly, recent evidence links an SDM program to fewer unplanned dialysis starts; generally, predialysis education and treatment planning allow patients to consider modality options and plan accordingly before dialysis becomes an emergency [28]. However, current evidence is insufficient to show that SDM alone affects survival. Age, frailty, comorbidity, nutrition, and functional status are significant and strong contributors to mortality in advanced CKD. Therefore, any survival difference between treatment groups should be interpreted in relation to individual patient characteristics, not directly as a result of the SDM intervention [28]. The SDM's main responsibility is to ensure the patient understands the risks and benefits of therapy and selects a course of action that aligns with their objectives.

SDM tools are a viable addition to advanced CKD care, as evidenced by the high acceptability of decision-support interventions and the enhanced dialog with physicians and family members. This is consistent with earlier studies showing that decision aids can enhance patient satisfaction and participation in treatment choices [27]. Because decisions on dialysis and CKM might impact caregiving, transportation, household duties, and the viability of home-based therapies, family engagement is especially important. Research indicates that while the patient's preferences should always come first, family and social support may significantly influence modality choices [21,24]. The psychological and peer-support needs identified in the present review further demonstrate that SDM should not focus exclusively on medical information. Patients approaching kidney failure may experience uncertainty, fear, grief, and concerns about loss of independence. Incorporating psychosocial and peer support may therefore strengthen SDM by helping patients process information and relate treatment options to their lived experiences.

The need to incorporate CKM into standard SDM is one of this review's most significant implications. The included studies' limited understanding of CKM aligns with earlier research showing that patients and medical providers may not adequately address conservative treatment [25,26]. A CKM-specific decision aid was found to be both practicable and acceptable in a randomized pilot study, which also encouraged conversations about CKM among patients, relatives, and medical professionals [29]. Therefore, rather than being portrayed as a lack of treatment, CKM should be offered alongside dialysis and transplantation as an active, customized treatment pathway. This is especially important for elderly or frail patients, whose quality of life, symptom burden, functional status, and personal priorities may all play a significant role in treatment decisions.

Overall, the findings support SDM as an important component of patient-centered care in advanced CKD. Its principal benefit appears to be improving the process and quality of decision-making, increasing knowledge, facilitating communication, and promoting treatment choices that better reflect patients' preferences. SDM should therefore be implemented early, repeated throughout disease progression, and supported by balanced decision aids, family involvement, and psychosocial support. Future research should focus not only on modality selection and survival but also on decision quality, treatment concordance, quality of life, decisional conflict, and long-term patient satisfaction.

Limitations and Strengths

This review has several limitations. Significant variation in SDM outcome measures, including the use of several tools such as SDM-Q-9, SAM, and concordance indicators, hindered cross-study comparability and made meta-analysis impossible. Most included research was from high-income settings, which may limit its applicability to resource-limited healthcare systems. Participants in SDM-focused studies may reflect more involved and health-literate people, which raises the possibility of selection bias. Furthermore, there was little data on clinical results, implementation challenges, and practicality; only a small number of studies reported outcomes related to tool usage and survival. Publication bias may have favored research showing favorable SDM outcomes, while cost-effectiveness statistics were completely lacking.

This review has notable strengths, including a comprehensive synthesis of evidence across multiple databases and study designs that captures a broad range of SDM-related outcomes. It integrates procedural, behavioral, clinical, and patient-reported outcomes to provide a comprehensive evaluation of SDM in advanced CKD. The inclusion of current studies (2020-2026) strengthens the findings’ present relevance. Furthermore, the review includes both quantitative and qualitative information, which improves interpretability across domains. Importantly, the emphasis on patient-centered outcomes beyond traditional clinical objectives aligns with contemporary priorities in chronic disease care.

Future Implications

The findings have important therapeutic implications for introducing structured SDM pathways in nephrology practice. These should include standardized patient information materials, tailored psychological support systems, and regular prognostic communication concerning illness progression and life expectancy. Evidence of improved treatment concordance supports SDM as a technique to minimize needless dialysis initiation and enhance care coordination. Future research should focus on developing validated CKD-specific SDM instruments, comparing the efficiency of different SDM models, and conducting longitudinal studies on quality of life and caregiver outcomes. Furthermore, implementation research and cost-effectiveness assessments are essential to ensure scaled adoption across multiple healthcare systems.

## Conclusions

This systematic review shows that using SDM in advanced CKD increases treatment concordance, patient engagement, and care alignment with patient preferences. However, SDM quality remains modest, despite significant gaps in prognostic communication, psychosocial support, and awareness of conservative therapeutic alternatives. Decision-support strategies have shown high acceptance and therapeutic benefit, particularly in minimizing unscheduled dialysis initiation and improving treatment planning. Despite these benefits, SDM adoption remains variable and insufficient across care settings. Overall, organized SDM models are effective, but they must be combined with psychosocial and communication-focused components to provide the best possible patient-centered care.

## Appendices

**Table 5:** Supplementary appendix

PubMed = 206	Embase = 335	Scopus = 116	Cochrane = 98
("kidney failure, chronic"[MeSH Terms] OR "renal insufficiency, chronic"[MeSH Terms] OR "chronic kidney disease"[Title/Abstract] OR "CKD"[Title/Abstract] OR "end stage renal disease"[Title/Abstract] OR "ESRD"[Title/Abstract] OR "advanced chronic kidney disease"[Title/Abstract] OR "kidney failure"[Title/Abstract]) AND ("shared decision making"[Title/Abstract] OR "patient participation"[MeSH Terms] OR "patient-centered care"[MeSH Terms] OR "decision making"[Title/Abstract]) AND ("Decision Support Techniques"[MeSH Terms] OR "decision support"[Title/Abstract] OR "decision aid*"[Title/Abstract] OR "decision tool*"[Title/Abstract] OR "decision model*"[Title/Abstract] OR "treatment decision*"[Title/Abstract]) AND ("dialysis"[Title/Abstract] OR "hemodialysis"[Title/Abstract] OR "peritoneal dialysis"[Title/Abstract] OR "transplantation"[Title/Abstract] OR "kidney transplantation"[MeSH Terms] OR "conservative management"[Title/Abstract] OR "supportive care"[Title/Abstract])	('chronic kidney failure'/exp OR 'chronic kidney disease'/exp OR 'end stage renal disease'/exp OR 'kidney failure' OR ckd OR esrd OR 'advanced chronic kidney disease') AND ('shared decision making'/exp OR 'shared decision making' OR 'patient participation'/exp OR 'patient centered care'/exp) AND ('decision support system'/exp OR 'decision support' OR 'decision aid' OR 'decision tool' OR 'clinical decision making'/exp) AND ('dialysis'/exp OR 'hemodialysis'/exp OR 'peritoneal dialysis'/exp OR 'kidney transplantation'/exp OR 'conservative treatment'/exp OR 'supportive care')	( TITLE-ABS-KEY ( "chronic kidney disease" OR CKD OR "end stage renal disease" OR ESRD OR "advanced kidney disease" OR "kidney failure" ) AND TITLE-ABS-KEY ( "shared decision making" OR "patient participation" OR "patient centered care" OR "decision making" ) AND TITLE-ABS-KEY ( "decision support" OR "decision aid" OR "decision tool" OR "decision model" OR "treatment decision" ) AND TITLE-ABS-KEY ( dialysis OR hemodialysis OR peritoneal dialysis OR transplantation OR "kidney transplant" OR "conservative management" OR "supportive care" ) )	#1 MeSH descriptor: [Renal Insufficiency, Chronic] explode all trees OR MeSH descriptor: [Kidney Failure, Chronic] explode all trees OR "chronic kidney disease":ti,ab,kw OR CKD:ti,ab,kw OR "end stage renal disease":ti,ab,kw OR ESRD:ti,ab,kw OR "advanced chronic kidney disease":ti,ab,kw OR "kidney failure":ti,ab,kw #2 MeSH descriptor: [Shared Decision Making] explode all trees OR "shared decision making":ti,ab,kw OR "patient participation":ti,ab,kw OR "patient-centered care":ti,ab,kw OR "decision making":ti,ab,kw #3 MeSH descriptor: [Decision Support Techniques] explode all trees OR "decision support":ti,ab,kw OR "decision aid*":ti,ab,kw OR "decision tool*":ti,ab,kw OR "decision model*":ti,ab,kw OR "clinical decision making":ti,ab,kw #4 dialysis OR hemodialysis OR peritoneal dialysis OR MeSH descriptor: [Renal Replacement Therapy] explode all trees OR MeSH descriptor: [Kidney Transplantation] explode all trees OR "kidney transplant*":ti,ab,kw OR "conservative management":ti,ab,kw OR "supportive care":ti,ab,kw #5 #1 AND #2 AND #3 AND #4
